# Differences of Various Region-of-Interest Methods for Measuring Dopamine Transporter Availability Using ^99**m**^ **T** **c**-TRODAT-1 SPECT

**DOI:** 10.1155/2014/837439

**Published:** 2014-07-01

**Authors:** Tang-Kai Yin, Bi-Fang Lee, Yen Kuang Yang, Nan-Tsing Chiu

**Affiliations:** ^1^Department of Computer Science and Information Engineering, National University of Kaohsiung, 700 Kaohsiung University Road, Kaohsiung 811, Taiwan; ^2^Department of Nuclear Medicine, National Cheng Kung University Hospital, College of Medicine, National Cheng Kung University, Tainan 704, Taiwan; ^3^Department of Psychiatry, National Cheng Kung University Hospital, College of Medicine, National Cheng Kung University, Tainan 704, Taiwan

## Abstract

This study was to investigate whether various region-of-interest (ROI) methods for measuring dopamine transporter (DAT) availabilities by single photon emission computed tomography (SPECT) are statistically different, whether results of medical research are thereby influenced, and causes of these differences. Eighty-four healthy adults with ^99m^Tc-TRODAT-1 SPECT and magnetic resonance imaging (MRI) scans were included. Six major analysis approaches were compared: (1) ROI drawn on the coregistered MRI; (2) ROIs drawn on the SPECT images; (3) standard ROI templates; (4) threshold-ROIs; (5) atlas-based mappings with coregistered MRI; and (6) atlas-based mappings with SPECT images. Using the atlas-based approaches we assessed the influence of striatum ROIs by slice-wise and voxel-wise comparisons. In (5) and (6), three partial-volume correction (PVC) methods were also explored. The results showed that DAT availabilities obtained from different methods were closely related but quite different and leaded to significant differences in determining the declines of DAT availability per decade (range: 5.95–11.99%). Use of 3D whole-striatum or more transverse slices could avoid biases in measuring the striatal DAT declines per decade. Atlas-based methods with PVC may be the preferable methods for medical research.

## 1. Introduction

Imaging the dopamine (DA) neurotransmission system using single photon emission computed tomography (SPECT) or positron emission tomography (PET) with various radioligands can provide quantitative information about the central dopaminergic system. Because DA modulates many vital physiological functions such as movement, motivation, cognition, reward, emotional behavior, and neuroendocrine regulation, the imaging techniques have wide and important applications in both medical research and clinical practice.

Analysis of DA neurotransmission SPECT or PET images acquired at an optimal imaging time point to obtain a simple ratio of specific to nonspecific binding is a validated method for assessing the DA neurotransmission system [1] and has been extensively used. Defining the region-of-interest (ROI) of the specific binding area (the striatum) and nonspecific binding area (reference areas, devoid of the DA neurotransmission system, such as the occipital area or cerebellum) is necessary for the ratio methods. Currently there are various methods in use. The ROIs can be delineated manually [2–4], using the threshold of maximal count within the striatal area [5], placing geometrically shaped ROIs [6], using templates [7, 8], or manually drawing on the coregistered MRI images [9–11]. Statistical parametric mapping (SPM; Wellcome Trust Centre for Neuroimaging), a voxel-based analysis, can also be used by normalizing the individual scans to the same Montreal Neurological Institute (MNI) template spatially to calculate the ratio of specific to nonspecific binding. Because the patterns of the DA neurotransmission system are different from those of an SPM8 ^99m^Tc-HMPAO SPECT template or an SPM99 ^15^O-H_2_O PET template, these standard templates provided with SPM cannot be directly applied [12]. Two approaches have usually been used. The first one is a direct approach in which the ligand-specific template is created with SPM [13, 14] or from other researches [15]. The second is an indirect approach in which MRI scans are spatially normalized to the standard MRI templates provided in SPM, and then the obtained transformation matrices are applied to the DA neurotransmission SPECT or PET [16]. These different ROI methods may or may not produce significantly different results using data from the same patients; therefore, it is desirable to know the basic differences between these methods. Few studies, however, have addressed this issue.

The spatial resolution of SPECT and PET is relatively low. The partial volume effect (PVE) may cause significant bias. The quantification values are affected by PVE such that many researchers have emphasized the importance of partial volume correction (PVC) [17–19]. However, considering the relative quantification values between patients, or for the same patient at different ages, PVC may not significantly improve relative quantification accuracy for classification results [20] or striatal declines of DAT availabilities per decade—relative quantities at different ages—in this study. Some studies have shown that PVC is not essential when quantifying the binding-potential differences between patients [21, 22].

In this study, we recruited healthy volunteers who underwent ^99m^Tc-TRODAT-1 SPECT and MRI scans. DAT availability was determined using different ROI quantification methods. The differences in the declines of DAT availabilities per decade between these methods and the causes of these differences were investigated. The effect of PVC was also explored in this study.

## 2. Materials and Methods

### 2.1. Participants

Eighty-four healthy adult volunteers (35 men, 49 women; mean age: 35.7 ± 12.5 years; range: 18.9–60.8 years) were recruited by advertising in a local newspaper. They were checked by a senior psychiatrist to exclude those with mental illness. Other exclusion criteria were pregnancy, any past or present neurological disorder, alcohol or substance abuse, and a history of head trauma with a loss of consciousness. In addition, all participants were given a brain MRI. Those with abnormal brain MRI findings were excluded. The included participants then underwent a ^99m^Tc-TRODAT-1 SPECT scan to assess DAT availability. Most of them had been enrolled in previous studies as healthy controls [8, 9]. The study protocol was approved by the Ethics Committee for Human Research of our institution, and informed consent was obtained from the volunteers before any procedure was performed.

### 2.2. Image Acquisition

Four hours after the participants had been given an intravenous injection of 740 MBq (20 mCi) of ^99m^Tc-TRODAT-1, the SPECT images were acquired using a triple-headed *γ*-camera with ultrahigh resolution fan beam collimators (Multispect 3; Siemens, Hoffman Estates, IL, USA), which yielded an image resolution of approximately 8.5 mm full-width at half-maximum. A dual-strip instant thin-layer chromatography method [23] was performed to ensure the radiochemical purity of ^99m^Tc-TRODAT-1 > 90%. The SPECT data were acquired over a 360° rotation in 120 steps, 50 s per step, in a 128 × 128 × 16 matrix. Reconstruction was done using a Butterworth filter (cutoff frequency: 0.4 Nyquist; power factor: 7). Attenuation correction was then applied using Chang's method [24]. The reconstructed transverse images were realigned parallel to the canthomeatal line. The slice thickness of each transverse image was 2.89 mm. Each participant also underwent a brain MRI (Signa CV-1, 1.5 tesla; GE Medical Systems, Milwaukee, WI, USA).

### 2.3. Quantitative Analysis of Striatal DAT Availability

The DAT availability was calculated as

(1) $$\begin{array}{c} \frac{\text{ST}-\text{OC}}{\text{OC}}, \end{array}$$

where ST and OC are the mean counts of the striatum and occipital areas, respectively. To study the biases or variances resulting from different ROI quantification methods, six major approaches similar to those in many other studies were explored.

*(1) MRI-Delineation.* Each participant's ^99m^Tc-TRODAT-1 SPECT image was automatically coregistered with the corresponding T2-weighted MRI image and then finely adjusted by an experienced nuclear medicine physician using a commercial software (PMOD; PMOD Technologies, Zurich, Switzerland). The MRI image was loaded as a reference. On two contiguous MRI transverse slices (thickness: 3.3 × 2 = 6.6 mm), the ROIs of the striatum and occipital areas were manually delineated and then used to project the MRI images onto the coregistered ^99m^Tc-TRODAT-1 SPECT images [9]. 

*(2) SPECT-Delineation.* The six consecutive SPECT transverse slices that best visualized the striatum were combined. ROIs were then manually drawn over the striatum and occipital areas on the ^99m^Tc-TRODAT-1 SPECT of each participant by an experienced nuclear medicine physician based on the individual MRI (nonregistered) [2]. 

*(3) Template-ROI.* A set of standard SPECT ROI templates that defined the striatum and occipital areas was first established. The six consecutive SPECT transverse slices that best visualized the striatum were then combined. An experienced nuclear medicine physician next manually positioned the templates on the transverse TRODAT-1 SPECT images of each participant without changing the size or shape of the ROIs [8]. 

**Figure 1:**
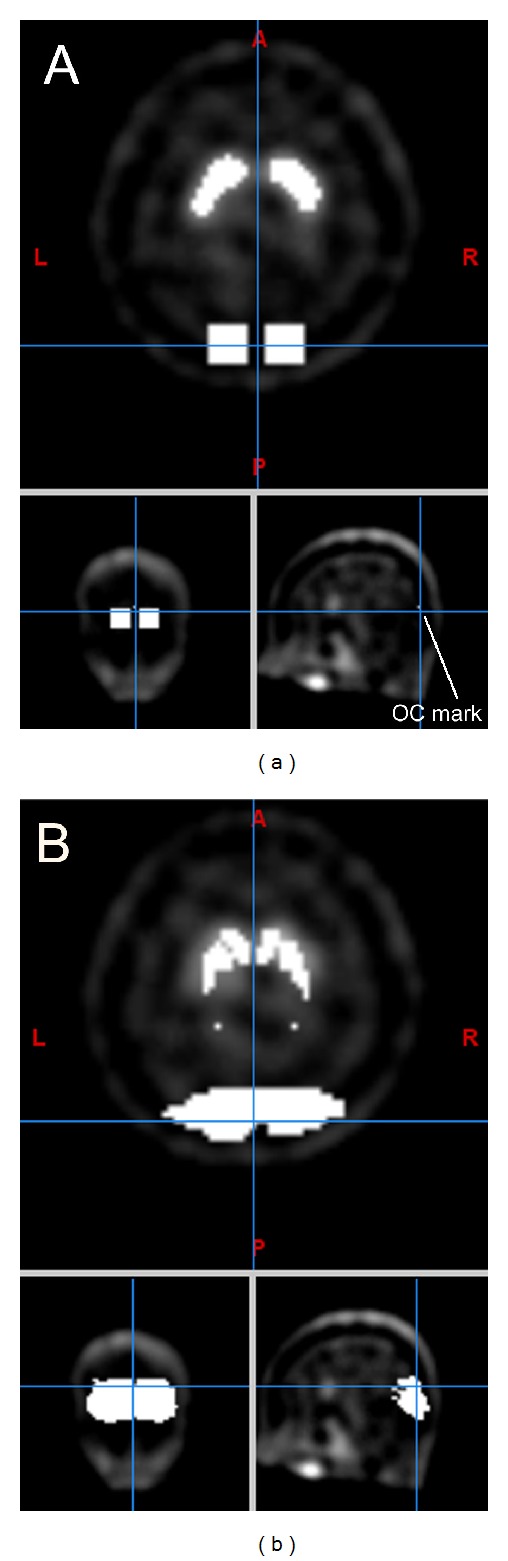
ROIs of striatum and occipital lobe in the TRODAT-1 SPECT of a 23-year-old woman (native space). (a) 70% threshold-ROI method and (b) SPECT-normalization approach.

*(4) Threshold-ROI.* Using a medical research image viewer (Mango; http://ric.uthscsa.edu/mango/mango.html), a single-voxel occipital mark (OM) in the central of brain (*x* axis), immediately before the skull (*y* axis), and at the transverse slice (*z* axis) with the largest striatum area was manually labeled for each participant. Denote the coordinate of OM as (OM_*x*_, OM_*y*_, OM_*z*_) in the voxels of the native space. We used codes that we created ourselves (MATLAB; MathWorks, Natick, MA, USA) to find the maximum count of the 21 transverse slices between OM_*z*_ − 10 and OM_*z*_ + 10, and then we located the two largest connected areas with counts larger than 90% of this maximum count on the ^99m^Tc-TRODAT-1 SPECT; one area (>OM_*x*_) overlapped with the right striatum and the other (<OM_*x*_) with the left striatum. The mean counts of these areas were denoted as ST. Then, OC was calculated as the averages of two 7 × 7 × 7 cubes centered at (OM_*x*_ − 5, OM_*y*_ + 6, OM_*z*_) and (OM_*x*_ + 5, OM_*y*_ + 6, OM_*z*_). These two centers were chosen after comparing and finding similar OC counts with those in the following atlas-based approaches. Other thresholds, 85%, 80%, 75%, 70%, 65%, and 60%, were also used (Figure 1(a)). 

*(5) Atlas-Based Mappings with Coregistered MRI (MRI-Normalization).* To automatically read the DAT availability, striatum and occipital masks from the WFU PickAtlas Standard Atlases [25, 26] in the Montreal Neurological Institute (MNI) space (http://www.nil.wustl.edu/labs/kevin/man/answers/mnispace.html) were used. The voxel positions (*x*: −90 : 2 : 90, *y*: −126 : 2 : 90, and *z*: −72 : 2 : 108) were the same as in the templates provided by SPM8. We spatially normalized the participants' MRI scans to the MRI template provided by SPM and collected from the original MRI scans the voxels that were mapped to the above WFU striatum and occipital masks in the MRI template. ^99m^Tc-TRODAT-1 SPECT images were coregistered to their MRI images using SPM coregistration default settings. The ROIs of the striatum and the occipital lobe in the SPECT images were defined as those voxels that were mapped to the collected voxels in the original MRI images. Finally, the DAT availabilities were obtained by calculating the averages of the striatum and occipital ROIs, respectively, in the SPECT images. 

*(6) Atlas-Based Mappings with SPECT Images (SPECT-Normalization).* Different from the MRI-normalization approach, a ligand-specific template has to be made before direct normalization is applied. We randomly selected 30 participants, spatially normalized their MRI scans to the MRI template provided by SPM, and kept the transformation parameters. Then the 30 ^99m^Tc -TRODAT-1 SPECT images were individually registered to their MRI images using the SPM coregistration default settings. These individually registered SPECT images were then spatially normalized into 91 × 109 × 91 images using the SPM spatial normalization default settings with the above individual transformation parameters. The creation of the following new template is an off-line fusion. These images were divided by their corresponding occipital averages, and then the voxel-wise averages of these 30 participants were saved as new images. We then used SPM spatial smoothing at 8 mm full-width at half maximum in *x*, *y*, and *z* directions to these images to obtain the ligand-specific template.

SPM was used to spatially normalize all the original SPECT images of the participants to this ligand-specific template. We collected the voxels in the original SPECT images which were mapped to the above WFU striatum and occipital masks in the ligand-specific template. With the derived striatum and occipital ROIs, the DAT availabilities were obtained (Figure 1(b)).

Our method is similar to a previous study [27], but we used SPM in automatic coregistration instead of manual one and the voxel-based analysis was not used here because our comparisons were focused on the averages of striatum instead of individual voxels. For lack of an available ^11^C-raclopride template, we did not make the template as Kas et al. [28].

### 2.4. Assessment of Influence of ROI Locations

Because the atlas-based approaches provided 3D whole-striatum ROIs both in the native and MNI spaces, the influence of ROI locations could be conveniently investigated by slice-wise and voxel-wise comparisons. 

*(a) Slice-Wise Comparisons.* Based on the coordinate OM_*z*_ of each participant, which denotes the transverse slice containing the largest striatum area, all SPECT scans could be averaged for all transverse scans at the same OM_*z*_ − 2,…, and OM_*z*_ + 6 coordinates and atlas-defined and threshold-defined striatum ROIs. Then slice-wise comparisons of the declines per decade could be made. 

*(b) Voxel-Wise Comparisons.* Because the voxels of all participants were registered on the MNI space in both the atlas-based approaches, the declines per decade of DAT availabilities at the voxel level could be assessed.

### 2.5. Partial Volume Correction

Both MRI- and SPECT-normalization methods were used with three partial volume correction (PVC) methods: the reblurred Van Cittert deconvolution (VC), the geometric transfer matrix (GTM) method, and the region-based voxel-wise (RBV) correction [17]. The VC method is a deconvolution-based image restoration method in which the three-dimensional point-spread function of the scanner is used to convolve with the estimated image at each iteration, and after convergence, the corrected images are obtained [19]. No anatomical information is needed with the VC method.

For the GTM and RBV methods, the brain is segmented into nonoverlapping ROIs that are assumed to be homogeneous. In this study, our focus was on the caudate and the putamen. Using the WFU atlases and Talairach atlas labels, 21 nonoverlapping ROIs were selected: 11 level-2 ROIs (posterior lobe, anterior lobe, frontal-temporal space, limbic lobe, medulla, pons, midbrain, occipital lobe, temporal lobe, parietal lobe, and frontal lobe) and 10 level-3 ROIs (insula, extra-nuclear, lentiform nucleus excluding putamen, claustrum, thalamus, fourth ventricle, third ventricle, lateral ventricle, caudate, and putamen). Using the mappings between ^99m^Tc-TRODAT-1 SPECT images and the normalization templates of the MNI, these 21 ROIs could be defined for each participant in the native space. Incorporating the geometric interactions between these 21 ROIs into linear equations and then solving them, the GTM method recovered the accuracy of multiple regions, especially of the caudate and putamen in the striatal DAT quantifications. In the RBV method, the obtained GTM-corrected values were used to calculate a voxel-wise correction of the entire image [17].

### 2.6. Statistical Analysis

To check the similarity in the obtained DAT availabilities, Pearson product-moment correlation coefficients (*r*) were first computed to check the linear associations, and then, the ratios of the root-mean-square difference to the average (normalized RMSD) were further calculated to study the agreement between any two quantification methods. To compare the age-related declines of DAT availability, a linear regression line was calculated for each quantification method using age as the regressor and DAT availability as the dependent variable. The declines in DAT availabilities per decade were calculated as $d=-10{\hat{\beta }}_{1}/({\hat{\beta }}_{0}+{\hat{\beta }}_{1}x_{\min})\times 100\%$ which was a combination of the estimated intercept ${\hat{\beta }}_{0}$, slope ${\hat{\beta }}_{1}$, and the minimal age in the participants, *x*_min⁡_. The bias-corrected and accelerated (BC_*a*_) percentile method with 10,000 bootstrap samples was used to find the 100 × (1 − *α*)% bootstrap confidence interval for the difference of a pair of declines per decade: *d* ^(*a*)^ − *d* ^(*b*)^ [29]. The value *α* was set at 0.05/(the number of pairs) using the simple Bonferroni correction of multiple tests. All statistical tests were performed by using EXCEL (Microsoft, Redmond, WA, USA) or MATLAB (MathWorks, Natick, MA, USA). Significance was set at *P* < 0.05.

## 3. Results

### 3.1. Linear Associations and Disagreement between the Quantification Methods

Totally, 18 ROI quantification methods were explored (Tables 1 and 2). The correlation coefficients (*r*) ranged between 0.47 (*P* = 5.7 × 10^−6^) and 1.00 (Table 1). Low values of the correlation coefficients exist between SPECT-delineation and the other methods (0.47 ≤ *r* ≤ 0.56). All the corresponding *P* values were <0.05, which indicated that the linear associations derived using these methods were significant and that the methods are closely related.

On the contrary, the maximum and minimum of the normalized RMSD were 83% and 4%, respectively (Table 1), which showed that there are large differences between these methods.

### 3.2. Detecting Age-Related Declines

All the slopes of these DAT quantification methods were <0 (*P* < 0.05 for all), which showed that the age-related declines were detected in all 18 methods. The slopes were between −0.011 (template-ROI) and −0.028 (MRI-normalization with GTM). Six of the 8 normalization methods showed more negative values of slopes than did the other methods; contrarily, the two manual methods, SPECT-delineation and template-ROI, had less negative values of slopes (Table 2).

### 3.3. Declines per Decade

Various ROI quantification methods resulted in large differences in DAT availability declines per decade, from 5.95% (90% threshold-ROI) to 11.99% (MRI-normalization with RBV). The smallest and the largest declines per decade were significantly different (*P* < 0.05, Bonferroni correction of multiple tests for 153 pairs, each having 10,000 bootstrap samples). The seven threshold-ROI methods and two manual methods (MRI-delineation, Template-ROI) yielded the smallest DAT availability declines per decade (5.95%–6.88%), followed by the four SPECT-normalization methods (7.99%–8.30%), and then the SPECT-delineation method (9.47%); the largest were the four MRI-normalization methods (11.02%–11.99%).

**Figure 2:**
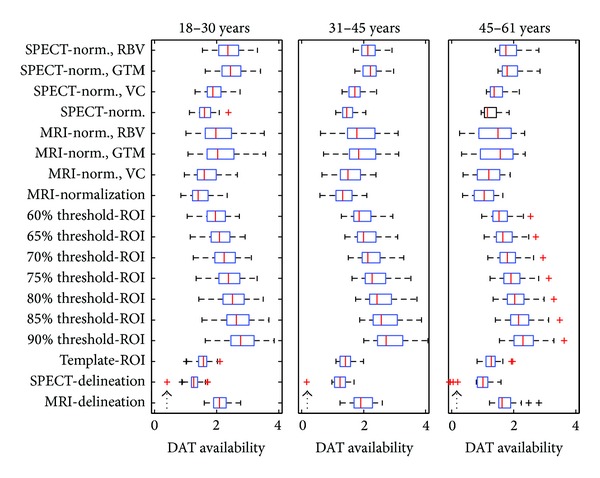
Boxplots of the three groups: 18–30, 31–45, and 46–61 years old, in the 18 methods of determining striatal DAT availability. The high decline of DAT availability per decade determined by SPECT-delineation method (9.47%) was partly caused by outliners. There were six outliners (arrowheads) in the SPECT-delineation method. Instead of a two-sided normal distribution of the outliners, all the six outliners were less than the mean. It might thereby lead to a high decline of DAT availability per decade.

From this ordering, if striatum was partly included such as two or six transverse slices in manual methods or threshold ROIs by 90%–60%, the declines per decade were smaller; otherwise, they were larger in 3D whole-striatum atlas-based methods. The only exception was the SPECT-delineation method (9.47%) in which the high decline of DAT availability per decade was partly caused by outliners because there were six outliners that all less than the mean—one-sided outliers instead of a two-sided normal distribution. This might be related to extra intra-operator errors (Figure 2, arrowheads).

### 3.4. Influence of ROI Locations Assessed in the Native Space by Slice-Wise Comparisons

For comparisons, the same OC averages from the threshold-ROI methods were used in (ST-OC)/OC in this subsection.  Table 3 listed the averages of striatal DAT availabilities, declines per decade of striatal DAT availabilities, and striatum sizes in the transverse slices at ≤(OM_*z*_) − 2, (OM_*z*_) − 1,…, and ≥(OM_*z*_) + 6 using the mapped 3D striatum ROIs from the atlas-based approaches. The maximal averages of striatal DAT availabilities were at (OM_*z*_) − 1 and gradually decreased as *z* axis increased, but the averages of striatal DAT availabilities from MRI-normalizations were lower than those from SPECT-normalizations (*P* = 9 × 10^−5^, paired *t*-test). The declines per decade increased from 5.12% to 14.42% (*r* = 0.99, *P* = 2.8 × 10^−7^ with *z* axis) for MRI-normalizations and from 4.15% to 12.70% and then 11.91% (*r* = 0.98, *P* = 3.5 × 10^−6^ with *z* axis) for SPECT-normalizations as *z* axis increased. The large differences across the *z* axis suggested that the 3D whole-striatum approaches or more transverse slices would be better choices.

For the average striatum sizes in the transverse slices, the maximum was at (OM_*z*_) because (OM_*z*_) was originally defined at the largest striatum transverse slice for each participant, while for MRI and SPECT normalizations, those were at (OM_*z*_) + 2 and (OM_*z*_) + 1, respectively. From Table 3 here, the declines per decade were 9.49% and 8.43%, which were different from 11.02% and 8.30% in Table 2, respectively, for MRI and SPECT normalizations; the differences of OC counts, two cubes versus atlas-defined ROIs, caused these differences.

Table 4 showed the differences between thresholds-ROIs and atlas-defined ROIs by calculating the declines per decade in the regions: (1) inside both the thresholds ROIs and the atlas-defined ROIs (*T*∩*A*), (2) inside the threshold ROIs but outside the atlas-defined ROIs (*T* − *A*), and (3) outside the threshold ROIs but inside the atlas-defined ROIs (*A* − *T*). The declines per decade were 5.04%–6.82% for *T*∩*A*, 6.00%–7.07% for *T* − *A*, and 7.59%–11.70% for *A* − *T*. The differences between them were significant: *P* = 0.018 between *T*∩*A* and *T* − *A*, *P* = 1.1 × 10^−8^ between *T*∩*A* and *A* − *T*, and *P* = 3.2 × 10^−8^ between *T* − *A* and *A* − *T*. The averages of *A* − *T*, 0.98–1.40, were much lower than those of *T*∩*A*, 1.97–2.71. Hence, a large part of striatum was not included in threshold-ROIs.

### 3.5. Influence of ROI Locations Assessed in the MNI Space by Voxel-Wise Comparisons

**Figure 3:**
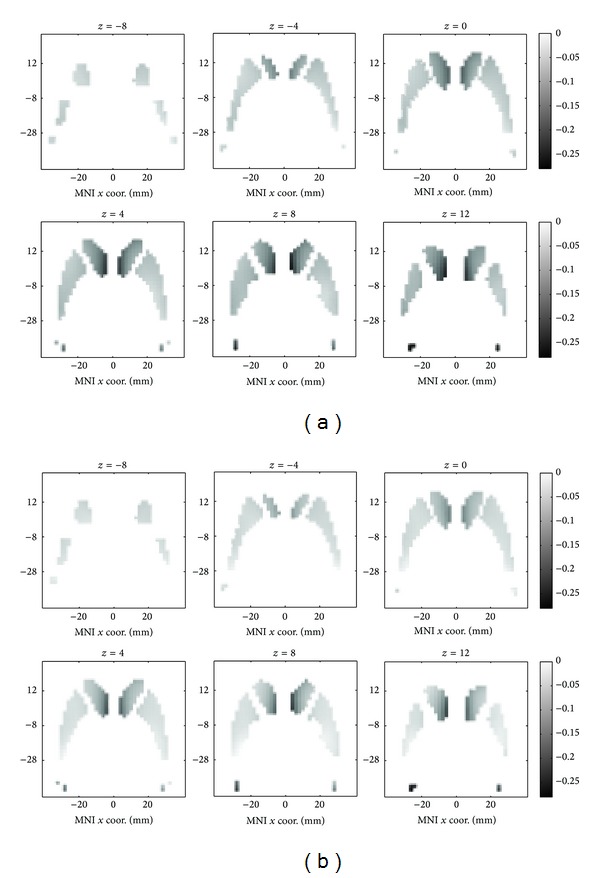
Decline per decade for each voxel in the MNI space. (a) MRI-normalization approach and (b) SPECT-normalization approach.

Figure 3 showed the declines per decade for each striatal voxel in the MNI space at six transverse slices. The variations were that (1) the declines per decade were larger at caudate than at putamen, (2) larger for MRI-normalizations than for SPECT-normalizations, and (3) those increased as the *z* axis increased. These variations over striatum showed that part-striatum ROIs would not be enough if their locations were not properly set.

### 3.6. PVC Comparisons

The pairwise Pearson product-moment correlation coefficients (Pearson's *r*) between these 4 methods ranged from 0.99 to 1 for MRI normalizations (Table 1); these high values indicate that they are closely related. Using the original, VC, and GTM as the regressors, *x*, respectively, and the RBV as the dependent variable, *y*, we have

(2) $$\begin{array}{c} y=-0.307+1.62x,\;R^{2}=0.985,\;\text{for}\;\text{the}\;\text{original},\\ y=-0.268+1.39x,\;R^{2}=0.984,\;\text{for}\;\text{the}\;\text{VC},\\ y=-0.072+1.00x,\;R^{2}=0.998,\;\text{for}\;\text{the}\;\text{GTM}. \end{array}$$

The near-one values of the coefficients of determination (*R* ^2^), which are the squares of Pearson's *r*, show that the PVC methods made corrections in linear proportions. Application of PVC caused higher DAT availabilities. For the average values, the VC method increased 14.9%, the GTM method 48.2%, and the RBV method 43.6%, but for the declines per decade, the VC method increased only 1.4%, the GTM 7.5%, and the RBV 8.8%. Regarding the declines per decade, the MRI-normalization with RBV here was the largest (11.99%; 95% CI: 8.45–15.03%) using 10,000 bootstrap samples (Table 2).

For SPECT-normalization and the three PVC methods, they were also closely related in linear regressions: *R* ^2^ was 0.958 for the original, 0.971 for the VC, and 0.996 for the GTM. The high *R* ^2^ values indicate that they were adequately described in linear regressions, which clearly modeled the corrections made by the three PVC methods. The averages of DAT availabilities increased 16.9%, 49.8%, and 44.8%, but the declines per decade decreased only 0.2% and decreased 3.7% and 3.1%, respectively, for the VC, GTM, and RBV methods.

## 4. Discussion

We investigated 18 ROI methods similar to the major methods to determine whether various methods for measuring DAT availability yielded different data,* namely*, DAT availability and DAT availability declines per decade, and, if so, what caused these differences. We found that using different methods yielded different DAT availability and thereby led to large differences in DAT availability declines per decade (from 5.95% to 11.99%). The age-related decline of striatal DAT availability has been reported as 3–10% per decade in healthy humans [30]. Therefore, our findings indicate that choosing an appropriate DAT analysis method is important.

ROIs can be delineated manually on SPECT scans [2–4], on the coregistered MRI images [9–11], or using templates [7, 8]. All these methods are hard to include the whole striatum. For example, ROI delineated manually on the coregistered MRI usually used only a few transverse slices of the highest striatal activities [9, 11] because delineating ROI is very tedious and time consuming. Our study showed that the declines of DAT availability per decade obtained from the above three methods from low to high were MRI-delineation (6.66%, using two slices), template-ROI (6.81%, using six slices), to SPECT-delineation (9.47%, using six slices). We found that using smaller ROIs (two versus six consecutive transverse slices) caused higher intercepts and smaller declines of DAT availability per decade.

The threshold-ROI methods can include high DAT-availability areas which are usually at or near striatum. We tried seven threshold values (90%, 85%, 80%, 75%, 70%, 65%, and 60%) in this study. The smaller ROIs (i.e., higher threshold) also results in the smaller declines of DAT availability per decade. However, the declines of DAT availability per decade (5.95–6.88%) yielded by the threshold-ROI methods were low. Because the threshold-ROI methods used only thresholds instead of the true striatum location, their results were biased to high DAT-availability areas. By comparing thresholds-ROIs and atlas-defined ROIs, we found that a large part of the striatum was not included in the threshold-ROIs.

We investigated two atlas-defined whole-stratum ROIs: MRI and SPECT normalization methods. While the MRI-normalizations used the MRI templates, the SPECT-normalizations used the ligand-specific normalization template. The declines of DAT availability per decade were lower for SPECT normalizations than for the MRI normalizations (8.04% versus 11.99% after RBV PVC). Although both methods were atlas-based analysis methods, the MRI structural information from which striatum ROIs were defined still resulted in differences.

The above findings illustrate that various ROI methods led to different results and these differences might result from the influence of ROI size, and location (including structural information). To further explore the causes, we performed the atlas-based approaches to assess the influence of striatum ROIs by slice-wise and voxel-wise comparisons. We observed that the variations inside the stratum were demonstrated in three ways: the declines of DAT per decade (1) increased as the *z* axis increased, (2) were larger in caudate than in putamen, and (3) were larger in low DAT-availability area than in high DAT-availability ones. These results indicate the importance of including the 3D whole-striatum or more transverse slices when determining DAT availability.

Both MRI and SPECT normalizations were tried with three PVC methods. The declines per decade were relative quantities and not significantly different between the original and the PVC methods. However, the averages of DAT availabilities were significantly larger after the PVC methods were used. If only the absolute quantification values were considered, the PVC methods were important [17–19]. There are a lot of PVC methods; among them, the VC and GTM were frequently tried in many researches and the RBV was a recent one demonstrating better results [17]. Hence, we tried these three PVC methods in this study.

It is difficult to determine the accuracy of DAT analysis methods. In this study, we investigated the effects of ROI sizes, locations and PVC on determining DAT availabilities and DAT availability declines per decade. Based on the results, we prefer the use of atlas-based methods with the PVC because (1) it provided a fully automatic method for labeling 3D whole-striatum-ROI at the voxel level, and (2) PVC recovered the degraded quantification values caused by the low spatial resolution of SPECT.

Several limitations of this study are listed as the follows. Firstly, compared to ^123^I-FP-CIT, the target-to-background ratio of ^99m^Tc-TRODAT-1 is lower [31]. The conclusions of this study may not be applicable to ^123^I-FP-CIT without experiments. Secondly, although SPM has been widely verified to be a useful voxel-wise analysis tool, the accuracies of coregistration and mapping to MNI templates from SPM may not be good enough for this study. Thirdly, the use of two and six transverse slices in the MRI and SPECT delineations, respectively, was based on the expertise of the operator; some other choice of the numbers of slices may be better for comparing with other methods. Finally, because there were no gold standards of DAT availability in this study, the accuracies of the tried methods were not available.

## 5. Conclusions

We evaluated 3 manual, 7 semiautomatic, and 8 automatic methods of obtaining striatum ROIs and compared declines per decade of striatal DAT availability between these methods. Declines per decade of striatal DAT availability determined using different methods were significantly different between manual, semiautomatic, and automatic methods. ROI size and location are important factors that cause these differences, which may affect the results of medical research. MRI-normalization and SPECT-normalization methods that consider the whole striatum may be more accurate. Using PVC recovered the degraded quantification values caused by the low spatial resolution of SPECT, and it influenced the results. For medical research, the atlas-based methods with PVC may be the preferable methods.

## Figures and Tables

**Table 1:** Pearson product-moment correlation coefficients (bottom left) and ratios of the root-mean-square differences to the averages (top right) between quantification methods of measuring DAT availabilities.

Method	1	2	3	4	5	6	7	8	9	10	11	12	13	14	15	16	17	18
(1) MRI-delineation	—	57%	33%	35%	30%	26%	21%	17%	15%	16%	43%	31%	23%	25%	30%	17%	19%	17%
(2) SPECT-delineation	0.56	—	32%	83%	79%	75%	70%	65%	59%	53%	32%	40%	61%	59%	35%	48%	70%	67%
(3) Template-ROI	0.79	0.65	—	62%	57%	52%	47%	42%	36%	30%	23%	23%	40%	38%	14%	23%	46%	43%
(4) 90% Threshold-ROI	0.74	0.50	0.70	—	5%	11%	17%	23%	30%	37%	71%	59%	40%	43%	59%	44%	21%	24%
(5) 85% Threshold-ROI	0.74	0.50	0.70	1.00	—	6%	11%	18%	25%	32%	66%	54%	36%	39%	54%	39%	17%	19%
(6) 80% Threshold-ROI	0.74	0.50	0.71	1.00	1.00	—	6%	12%	19%	26%	61%	50%	32%	35%	49%	34%	13%	15%
(7) 75% Threshold-ROI	0.74	0.51	0.71	1.00	1.00	1.00	—	6%	13%	21%	56%	45%	28%	31%	44%	29%	12%	12%
(8) 70% Threshold-ROI	0.75	0.51	0.72	1.00	1.00	1.00	1.00	—	7%	14%	51%	39%	25%	28%	38%	23%	13%	12%
(9) 65% Threshold-ROI	0.75	0.51	0.72	1.00	1.00	1.00	1.00	1.00	—	7%	45%	34%	24%	25%	31%	18%	17%	15%
(10) 60% Threshold-ROI	0.76	0.52	0.73	1.00	1.00	1.00	1.00	1.00	1.00	—	38%	28%	24%	25%	25%	13%	23%	20%
(11) MRI-normalization	0.78	0.55	0.70	0.74	0.74	0.74	0.74	0.75	0.75	0.75	—	14%	39%	36%	18%	31%	55%	51%
(12) MRI-norm., VC	0.78	0.55	0.70	0.73	0.73	0.73	0.74	0.74	0.75	0.75	1.00	—	26%	23%	15%	21%	43%	39%
(13) MRI-norm., GTM	0.75	0.52	0.68	0.71	0.71	0.71	0.71	0.72	0.73	0.73	0.99	0.99	—	4%	34%	23%	25%	23%
(14) MRI-norm., RBV	0.74	0.51	0.67	0.71	0.71	0.71	0.71	0.72	0.73	0.73	0.99	0.99	1.00	—	32%	22%	28%	26%
(15) SPECT-norm.	0.83	0.54	0.76	0.85	0.85	0.85	0.85	0.86	0.86	0.87	0.91	0.91	0.89	0.88	—	16%	42%	38%
(16) SPECT-norm., VC	0.82	0.52	0.75	0.85	0.85	0.85	0.85	0.86	0.86	0.87	0.88	0.88	0.86	0.86	1.00	—	26%	23%
(17) SPECT-norm., GTM	0.80	0.47	0.74	0.84	0.84	0.84	0.84	0.85	0.85	0.85	0.84	0.85	0.83	0.82	0.98	0.99	—	4%
(18) SPECT-norm., RBV	0.80	0.47	0.73	0.84	0.85	0.85	0.85	0.86	0.86	0.86	0.86	0.86	0.85	0.84	0.98	0.99	1.00	—

DAT: dopamine transporter; MRI: magnetic resonance imaging; SPECT: single photon emission-computed tomography; ROI: region of interest; norm.: normalization; GTM: geometric transfer matrix; RBV: region-based voxel-wise; VC: Van-Cittert deconvolution.

**Table 2:** Comparison of the regression lines between quantification methods of measuring DAT availabilities.

Method	Slope (*P* value∗)	Intercept	Decline per decade (95% CI∗∗)	*R* ^2^
(1) MRI-delineation	−0.015 (5.4 × 10^−6^)	2.49	6.66% (4.34–8.52%)	0.22
(2) SPECT-delineation	−0.013 (1.7 × 10^−5^)	1.62	9.47% (5.99–14.13%)	0.20
(3) Template-ROI	−0.011 (1.3 × 10^−5^)	1.86	6.81% (4.52–8.77%)	0.25
(4) 90% Threshold-ROI	−0.018 (1.7 × 10^−4^)	3.31	5.95% (3.34–8.10%)	0.16
(5) 85% Threshold-ROI	−0.017 (1.5 × 10^−4^)	3.15	6.09% (3.46–8.29%)	0.16
(6) 80% Threshold-ROI	−0.017 (1.4 × 10^−4^)	2.99	6.19% (3.63–8.45%)	0.16
(7) 75% Threshold-ROI	−0.016 (1.3 × 10^−4^)	2.84	6.29% (3.60–8.63%)	0.16
(8) 70% Threshold-ROI	−0.015 (1.0 × 10^−4^)	2.68	6.47% (3.45–8.73%)	0.17
(9) 65% Threshold-ROI	−0.015 (8.7 × 10^−5^)	2.52	6.64% (3.62–9.10%)	0.17
(10) 60% Threshold-ROI	−0.014 (7.9 × 10^−5^)	2.36	6.88% (3.86–9.42%)	0.17
(11) MRI-normalization	−0.018 (5.1 × 10^−8^)	1.95	11.02% (7.98–13.66%)	0.31
(12) MRI-normalization with VC	−0.021 (5.5 × 10^−8^)	2.24	11.17% (8.12–13.80%)	0.30
(13) MRI-normalization with GTM	−0.028 (1.7 × 10^−7^)	2.89	11.85% (8.45–14.77%)	0.29
(14) MRI-normalization with RBV	−0.027 (3.8 × 10^−7^)	2.80	11.99% (8.45–15.03%)	0.27
(15) SPECT-normalization	−0.014 (3.3 × 10^−9^)	2.01	8.30% (6.15–9.90%)	0.35
(16) SPECT-normalization with VC	−0.017 (4.5 × 10^−9^)	2.35	8.28% (6.18–9.93%)	0.34
(17) SPECT-normalization with GTM	−0.021 (1.2 × 10^−8^)	3.01	7.99% (5.84–9.63%)	0.33
(18) SPECT-normalization with RBV	−0.020 (2.0 × 10^−8^)	2.91	8.04% (5.87–9.71%)	0.32

CI, confidence interval; DAT, dopamine transporter; MRI, magnetic resonance imaging; SPECT, single photon emission-computed tomography; ROI, region of interest; GTM, geometric transfer matrix; RBV, region-based voxel-wise; VC, Van-Cittert deconvolution.

*P-values for *H*_0_: *β*_1_ = 0.

**95% Confidence intervals for declines per decade using 10,000 bootstrap samples.

**Table 3:** Striatal DAT availabilities, declines per decade of DAT availabilities, and striatum sizes in the transverse slices of the striatum ROIs defined by the atlas approaches.

Transverse slices∗∗	MRI-normalization			SPECT-normalization		
	Average∗∗∗	Decline per decade	Average size∗	Average∗∗∗	Decline per decade	Average size∗
≤(OM_*z*_) − 2	1.46	5.12%	68.4	1.54	4.15%	78.3
(OM_*z*_) − 1	1.78	6.19%	57.7	1.86	5.74%	67.4
(OM_*z*_)	1.76	7.67%	76.4	1.81	7.17%	87.2
(OM_*z*_) + 1	1.64	8.30%	89.8	1.66	8.04%	97.1
(OM_*z*_) + 2	1.44	9.41%	92.7	1.47	8.89%	94.3
(OM_*z*_) + 3	1.21	10.34%	87.5	1.25	10.29%	80.7
(OM_*z*_) + 4	0.99	11.56%	73.8	1.04	11.51%	60.8
(OM_*z*_) + 5	0.79	11.84%	56.9	0.85	12.70%	41.2
≥(OM_*z*_) + 6	0.58	14.42%	77.4	0.68	11.91%	30.4

*Voxels: each voxel is of 2.897 mm × 2.897 mm × 2.897 mm.

**OM_*z*_: the *z* axis of the OC mark is at the transverse slice with the largest striatum area.

***For comparisons, the same OC averages from the threshold-ROI methods were used in the calculation of (ST-OC)/OC.

**Table 4:** Striatal DAT availabilities, declines per decade of DAT availabilities, and striatum sizes in the regions: (1) inside both the threshold ROIs and the atlas-defined ROIs, (2) inside the threshold ROIs but outside the atlas-defined ROIs, and (3) outside the threshold ROIs but inside the atlas-defined ROIs.

Thresholds	Inside threshold, inside atlas			Inside threshold, outside atlas			Outside threshold, inside atlas		
	Average∗∗∗∗	Decline∗∗∗	Size∗∗	Average∗∗∗∗	Decline∗∗∗	Size∗∗	Average∗∗∗∗	Decline∗∗∗	Size∗∗
90%	2.71, 2.68∗	5.04%, 5.93%	33.8, 41.1	2.65, 2.62	6.00%, 6.10%	15.8, 8.5	1.28, 1.40	9.43%, 7.59%	646.8, 596.2
85%	2.55, 2.56	6.13%, 6.06%	69.4, 82.2	2.50, 2.48	6.11%, 6.23%	42.0, 29.1	1.22, 1.33	9.54%, 7.97%	611.3, 555.1
80%	2.43, 2.43	6.28%, 6.08%	113.4, 130.8	2.36, 2.33	6.17%, 6.43%	84.6, 67.2	1.14, 1.24	9.77%, 8.28%	567.2, 506.6
75%	2.31, 2.32	6.40%, 6.17%	162.2, 184.1	2.21, 2.19	6.27%, 6.47%	150.4, 128.5	1.06, 1.15	10.03%, 8.75%	518.4, 453.3
70%	2.19, 2.20	6.54%, 6.21%	215.9, 242.0	2.07, 2.04	6.46%, 6.68%	246.6, 220.5	0.96, 1.05	10.51%, 9.30%	464.7, 395.4
65%	2.08, 2.10	6.68%, 6.25%	270.0, 298.8	1.92, 1.89	6.64%, 6.84%	377.0, 348.2	0.87, 0.95	11.02%, 9.99%	410.6, 338.5
60%	1.97, 2.00	6.82%, 6.29%	325.4, 354.7	1.77, 1.75	6.90%, 7.07%	550.6, 521.3	0.78, 0.85	11.70%, 10.79%	355.2, 282.6

*In each pair of data, the left is for MRI-normalization, while the right is for SPECT-normalization.

**Size: each voxel is of 2.897 mm × 2.897 mm × 2.897 mm.

***Decline per decade.

****For comparisons, the same OC averages from the threshold-ROI methods were used in the calculation of (ST-OC)/OC.
